# Exploring the relationship between physical activity and cognitive function: an fMRI pilot study in young and older adults

**DOI:** 10.3389/fpubh.2024.1413492

**Published:** 2024-07-18

**Authors:** Jie Feng, Huiqi Song, Yingying Wang, Qichen Zhou, Chenglin Zhou, Jing Jin

**Affiliations:** ^1^Department of Sports Science and Physical Education, The Chinese University of Hong Kong, Shatin, Hong Kong SAR, China; ^2^The Jockey Club School of Public Health and Primary Care, The Chinese University of Hong Kong, Shatin, Hong Kong SAR, China; ^3^School of Psychology, Shanghai University of Sport, Shanghai, China; ^4^Key Laboratory of Exercise and Health Sciences of Ministry of Education, Shanghai University of Sport, Shanghai, China

**Keywords:** physical activity, healthy older adults, cognitive function, inhibitory control, brain activation

## Abstract

**Background:**

There are limited studies exploring the relationship between physical activity (PA), cognitive function, and the brain processing characteristics in healthy older adults.

**Methods:**

A total of 41 participants (42.7 ± 20.5 years, 56.1% males) were included in the data analysis. The International Physical Activity Questionnaire Short Form was used to assess PA levels, and the Chinese version of the Montreal Cognitive Assessment-Basic and the Flanker task were employed to evaluate cognitive function. Furthermore, fMRI technology was utilized to examine brain activation patterns.

**Results:**

The cognitive function of the older adults was found to be significantly lower compared to the young adults. Within the older adults, those with high levels of PA exhibited significantly higher cognitive function than those with low and medium PA levels. The fMRI data showed significant differences in brain activation patterns among young adults across the different PA levels. However, such difference was not observed among older adults.

**Conclusion:**

A decline in cognitive function was observed among older adults. There was a significant correlation between the levels of PA and cognitive function in healthy older adults. The study demonstrated significant effects of PA levels on brain activation patterns in inhibitory control-related regions among young adults, while not significant among older adults. The findings suggest that neurological mechanisms driving the relationship between PA and cognitive function may differ between older and young adults.

## 1 Introduction

The global population is currently experiencing an unprecedented phenomenon of rapid aging. From 2019 to 2030, the number of older adults is expected to rise from 1 billion to 1.4 billion (1). This demographic shift is occurring alongside an increasing prevalence of neurodegenerative diseases (e.g., cognitive decline, mild cognitive impairment, and Alzheimer’s disease), which are closely associated with aging. Cognitive problems can lead to a decline in the daily functioning and independence of older adults, which significantly increases the risk of institutionalization and the burden on caregivers (2). Given these circumstances, maintaining cognitive function becomes a major challenge for many individuals in older age (3).

Physical activity (PA) is a promising non-pharmacological intervention to maintain and improve brain structure and cognitive function across the lifespan (4). The physiological mechanisms suggest that PA can improve the capacity and speed of human cerebral blood flow to promote oxygenation in brain regions associated with cognitive function (5, 6). Engaging in PA during adulthood promotes cognitive development and slows the rate of cognitive decline (7). Several studies have suggested that PA level was positively associated with cognition in older adults. Specifically, a longitudinal study examined the dose–response association between PA and cognitive function, and the results showed that a higher level of PA was associated with a 36% lower risk of cognitive impairment and better maintenance of memory and executive function over time (8). De Souto Barreto et al. (9) found that compared with the inactive category, individuals in high-active, intermediate-active, and low-active categories had positive associations with cognitive function. Another cross-sectional study suggested that older adults participating in PA performed better in cognition than the no-physical-activity group (10). However, the relationship between PA and cognitive function in older adults at the level of brain structure and function is not yet well established (11).

Cognitive decline is associated with alterations to brain structure and function in older adults (12). Recently, functional structural and functional magnetic resonance imaging (fMRI) has been used to explore the link between PA and cognitive function in older adults. The fMRI is a non-invasive neuroimaging technique that provides a high spatial resolution, allowing for the detection of activity levels and connectivity patterns in specific brain regions (13). This makes fMRI well-suited to help understand the brain networks associated with cognitive function. By detecting the brain’s activity while performing a task, fMRI enables to understand the differences in neural functioning between different cognitive processes (14). Importantly, fMRI can also reveal how PA may influence brain function, providing insights into the underlying mechanisms by which PA may impact cognitive function (15). A systematic review found that the effects of aerobic exercise and fitness in patients with mild cognitive impairment and Alzheimer’s disease occur primarily in brain structures sensitive to neurodegeneration, including frontal, temporal, and parietal regions (15). However, there is limited study to examine the relationship between PA, cognitive function, and brain function in healthy older adults. Understanding the relationship between PA and cognitive function using fMRI can provide insights into the underlying neural mechanisms by which PA influences cognitive processes. This could inform the potential importance of PA and early interventions to delay the progression of neurodegenerative diseases. Therefore, this study aims to adopt high spatial resolution fMRI to explore the relationship between PA, cognitive function, and the distinct patterns of brain activation among a sample of healthy participants.

## 2 Materials and methods

### 2.1 Participants

This cross-sectional study was conducted among adults from a community center located in Liaoning Province, China. Utilizing a snowball sampling approach, initial participants were identified through social media advertisements and posters, and were then encouraged to refer additional eligible individuals from their personal networks. A total of 51 participants were included, including 22 young adults and 31 older adults. Inclusion criteria comprised individuals who were (1) adults aged ≥18 years; (2) had more than 10 years of education; (3) achieved a Montreal Cognitive Assessment Basic (MOCA-B) score higher than 19; (4) were in good health without visual, auditory, or communication impairments; and (5) reported no physical or mental illnesses. Participants were excluded if they (1) exhibited significant signs of cognitive decline, such as Alzheimer’s disease; (2) had a history or clinical manifestation of neurological or psychiatric conditions, such as brain tumors, severe depression, or schizophrenia; (3) were currently undergoing chemotherapy/radiation therapy; and (4) or had disabilities or difficulties in hearing, vision, or communication. Ethics approval for the study was granted by the Research Ethics Committee of Shanghai University of Sport, China. Written consent was obtained from all participants before commencing the study.

### 2.2 Montreal Cognitive Assessment

The MOCA-B was used as a cognitive screening tool to identify mild cognitive impairment in participants. The Chinese version of MOCA-B has demonstrated satisfactory reliability and validity among Chinese adults (16). Comprising 30 items, MOCA-B assesses various dimensions including (1) visuospatial and executive functioning, (2) naming, (3) attention (e.g., simple attention, working memory, vigilance), (4) language (e.g., repetition, phonemic fluency), (5) abstraction, (6) delayed memory recall, and (7) orientation. The total score, ranging from 0 to 30, was obtained by summing up all items, with a higher score indicating a higher level of cognitive function.

### 2.3 Physical activity

Participants’ PA was self-reported using the Chinese version of the International Physical Activity Questionnaire-Short Form (IPAQ-SF), encompassing walking, moderate-intensity PA (MPA), and vigorous-intensity PA (VPA). Following the guidelines for data processing and analysis of the IPAQ-SF (17), the total PA metabolic equivalent (MET)-minutes per week were computed by summing walking (3.3 MET), MPA (4.0 MET), and VPA (8.0 MET). Participants were categorized into two groups: (1) physically active, defined as achieving at least 3,000 MET-minutes per week; and (2) physically inactive, indicating less than 3,000 MET-minutes per week.

### 2.4 Cognitive function

Inhibitory control was measured using Eriksen flanker task (18). The stimuli consist of five horizontally arranged arrows, in two conditions: congruent and incongruent. In the congruent condition, all arrows faced in the same direction (> > > > >, or < < < < <); in the incongruent condition, the middle arrow faced in the opposite direction with other array (< < > < <, or > > < > >). A total of 100 randomized and equiprobable trials were presented. Each trial began with a presentation of array stimuli for 1,000 ms, followed by a response window and a jittered inter-trial-interval ranging from 2,000 to 6,000 ms, with intervals of 500 ms. Participants were instructed to respond quickly and accurately upon the appearance of the stimuli: index finger for a leftward middle arrow and middle finger for a rightward middle arrow. The tasks were performed using a fully randomized event-related design with a total duration of 414 s. The recreation time and accuracy (% correct) were measured, indicating inhibitory control and information processing, respectively. Also, the accuracy expressed as congruent minus incongruent condition, and recreation time expressed as incongruent minus congruent condition were computed (19). Data collection was conducted using E-prime 3.0 software (Psychology Software Tools, Inc.), which recorded participants’ accuracy and reaction times under both congruent and incongruent conditions. Trials with reaction times exceeding 2,800 ms or less than 100 ms were excluded. Due to thorough practice sessions prior to the formal scan, no trials were excluded.

### 2.5 Covariates

Gender, education level, height, and weight were self-reported by participants using a series of questions. Body mass index (BMI) was presented in weight (kg)/height squared (m^2^).

### 2.6 fMRI scanning parameters

Images were acquired using the GE Discovery MR-750 3.0T scanner (GE Discovery MR-750 3.0T scanner, GE Medical Systems, Waukesha, WI, USA). Participants were instructed to refrain from carrying any metallic objects and to minimize head movement. High-resolution three-dimensional T1-weighted structural images were obtained using a fast spoiled gradient-recalled echo (FSPGR) sequence, comprising 176 slices with a slice thickness of 1 mm and no gap, resulting in a voxel size of 1 × 1 × 1 mm^3^. The parameters were as follows: repetition time (TR) = 8.156 ms, echo time (TE) = 3.18 ms, flip angle = 8°, and field of view = 256 × 256 mm^2^. Functional images were acquired using a gradient-echo echo-planar imaging sequence, comprising 43 slices with a slice thickness of 3.2 mm and no gap, resulting in a voxel size of 3.44 × 3.44 × 3.2 mm^3^. The parameters were as follows: TR = 2,000 ms, TE = 30 ms, flip angle = 90°, and field of view = 220 × 220 mm^2^.

### 2.7 Statistical analysis

The variables were presented as mean ± standard deviation (SD) for continuous data, and as number and percentage of participants for categorical data. After adjusting for sex and education level, two-way ANOVA (PA level: low-to-moderate, high; age: young adults; older adults) followed by the Bonferroni post-hoc test were conducted to evaluate difference between PA levels and age group in cognitive function. All statistical analyses were conducted using SPSS version 27.0. The significance level was set at 0.05.

### 2.8 fMRI analysis

The data of fMRI were preprocessed using the Data Processing Assistant for Resting-State fMRI (DPARSF) toolbox,^1^ which included slice timing correction, head motion correction, normalization to the Montreal Neurological Institute (MNI) space, and resampling to a voxel size of 3 × 3 × 3 mm^3^. Participants with head motion greater than 2 mm were excluded from the final analysis. The normalized images were then smoothed using a Gaussian kernel with a full width at half maximum (FWHM) of 6 × 6 × 6 mm^3^.

After preprocessing, further analysis was conducted using Statistical Parametric Mapping (SPM8).^2^ First-level analysis was performed for each participant using a general linear model to estimate the contrasts of interest based on the experimental design (e.g., differences between conditions in the Flanker). Second-level analysis involved independent sample *t*-tests to compare the contrasts of interest between groups (i.e., older adults vs. young adults) for each task. Additionally, independent *t*-tests were conducted within each group to assess whole-brain activation using 0 as the reference value. Multiple comparison correction was applied using Gaussian random field (GRF) theory with voxel-wise thresholding set at *p* < 0.01 and cluster-wise thresholding set at *p* < 0.05.

Based on the second-level analysis results, clusters of activation were identified, and regions of interest (ROIs) were defined as spheres with a radius of 6 mm centered at the peak voxel within each cluster. To further explore the relationship between PA and brain activation, and to mitigate the impact of significant variations in individual PA data on the correlation analysis, Pearson correlation coefficients were then calculated to examine the relationships between behavioral measures (e.g., reaction time, accuracy) and imaging indices (contrasts of interest for each condition) within each group (young adults, older adults). Statistical significance was set at *p* < 0.05, and marginal significance *p* < 0.1.

## 3 Results

### 3.1 Participant characteristics

Among 52 participants, 11 participants were excluded due to incomplete questionnaires (*n* = 1) or incomplete fMRI scans (*n* = 10). Finally, a total of 41 participants (42.7 ± 20.5 years, 56.1% males) were included in the data analysis. Table 1 shows the characteristics of the participants. Among the 21 young adults (23.0 ± 2.1 years), 47.6% fell into the physically inactive category. In the group of 20 older adults (63.3 ± 2.4 years, 55.0% males), 40% were classified into the physically inactive. There was no significant difference between included and excluded participants, except for age; excluded participants had a higher age compared to included participants (64.3 ± 2.1 vs. 42.6 ± 20.5 years, *p* < 0.001).

**Table 1:** Participant characteristics (*n* = 41).

Characteristic	Mean ± SD or *n* (%)			*p* value (young vs. older adults)
	Whole sample	Young adults (*n* = 21)	Older adults (*n* = 20)	
Age (years)	42.7 ± 20.5	23.0 ± 2.1	63.3 ± 2.4	<0.001
Sex, *n* (%)				
Male	23 (56.1%)	12 (57.1%)	11 (55.0%)	0.890
Female	18 (43.9%)	9 (42.9%)	9 (45.0%)	
Height	169.83 ± 7.38	171.86 ± 7.40	167.70 ± 6.90	0.071
Weight	69.16 ± 11.14	65.52 ± 11.17	72.98 ± 10.00	0.030
Body mass index	23.94 ± 3.31	22.10 ± 2.91	25.87 ± 2.55	<0.001
Physical activity level, *n* (%)				
Physically inactive	18 (43.9%)	10 (47.6%)	8 (40.0%)	0.623
Physically active	23 (56.1%)	11 (52.4%)	12 (60.0%)	
Flanker_accuracy				
Congruent	0.98 ± 0.05	0.99 ± 0.03	0.97 ± 0.07	0.168
Incongruent	0.94 ± 0.14	0.98 ± 0.03	0.90 ± 0.20	0.079
Con–Incon	0.04 ± 0.14	0.01 ± 0.02	0.07 ± 0.19	0.168
Flanker_reaction time				
Congruent	594.29 ± 88.37	546.86 ± 57.92	644.10 ± 88.39	<0.001
Incongruent	685.30 ± 113.92	630.63 ± 61.85	742.71 ± 128.53	0.002
Con–Incon	91.01 ± 62.94	83.76 ± 36.13	98.61 ± 82.75	0.457

### 3.2 Age, physical activity, and cognitive function

Table 2 presents the accuracy and reaction time of the Flanker task for the young and older adults at different PA levels. For young adults, the accuracy for congruent trials was significantly higher than that for incongruent trials in the inactive group, compared to the active group (*p* < 0.05). However, there were no significant differences in other measures (*p* > 0.05). For older adults, the accuracy for incongruent trials in the active group was significantly higher than that in the inactive group (*p* < 0.05). Additionally, the accuracy for congruent minus incongruent trials and the reaction time for incongruent trials in the active group were significantly lower than those in the inactive group (*p* < 0.05). Again, there were no significant differences in other measures (*p* > 0.05).

**Table 2:** Behavioral results of the Flanker task for subjects at different physical activity levels.

	Young adults			Older adults		
	Physically inactive	Physically active	*p* value	Physically inactive	Physically active	*p* value
Accuracy						
Congruent	0.99 ± 0.03	0.99 ± 0.02	0.683	0.97 ± 0.04	0.97 ± 0.08	0.871
Incongruent	0.97 ± 0.04	0.99 ± 0.02	0.123	0.78 ± 0.28	0.97 ± 0.05	0.026
Con–Incon	0.02 ± 0.02	0.00 ± 0.01	0.019	0.19 ± 0.26	−0.01 ± 0.04	0.017
Reaction time (ms)						
Congruent	528.72 ± 63.09	563.35 ± 49.99	0.177	669.22 ± 117.65	627.35 ± 62.61	0.312
Incongruent	629.96 ± 78.01	631.23 ± 46.62	0.964	811.24 ± 175.91	697.02 ± 56.12	0.048
Con–Incon	−101.24 ± 40.11	−67.87 ± 24.07	0.075	−142.01 ± 113.36	−69.68 ± 36.96	0.530

### 3.3 fMRI results

**Figure 1:**
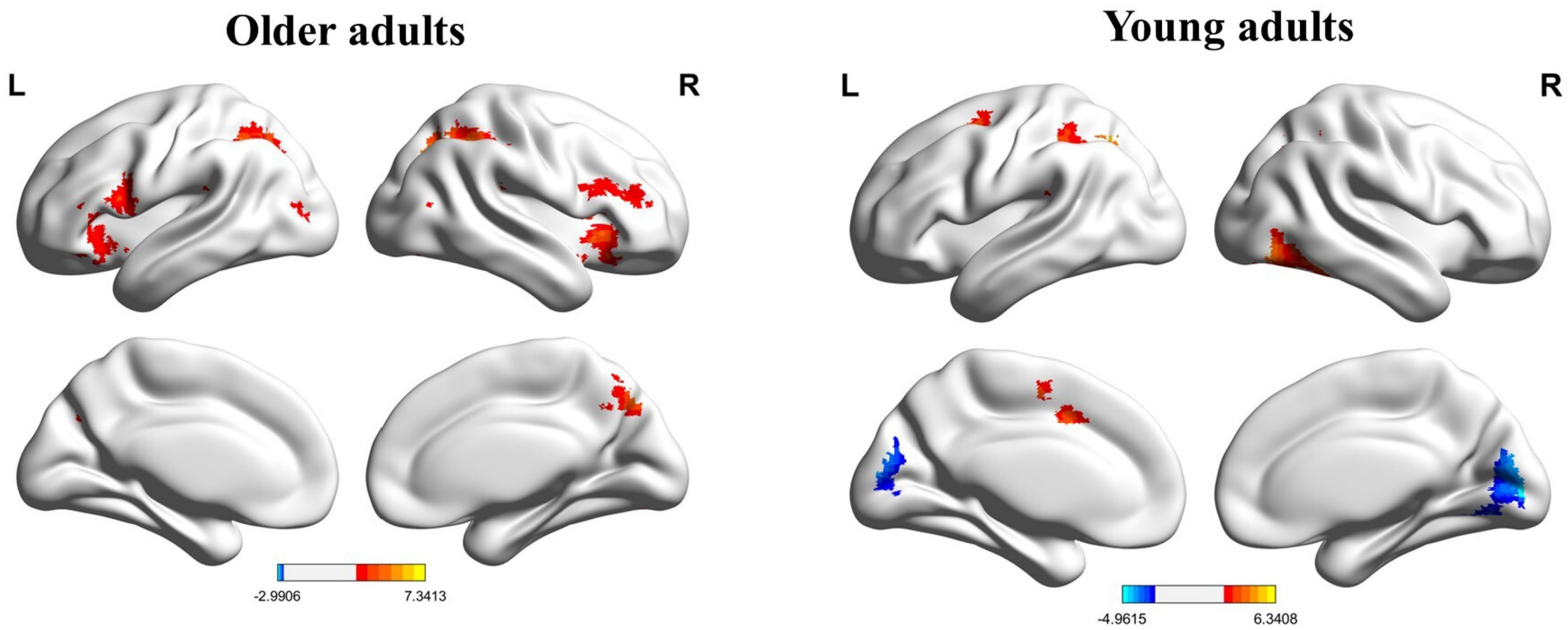
Whole brain activation maps of the older and young adults. L, left; R, right.

**Figure 2:**
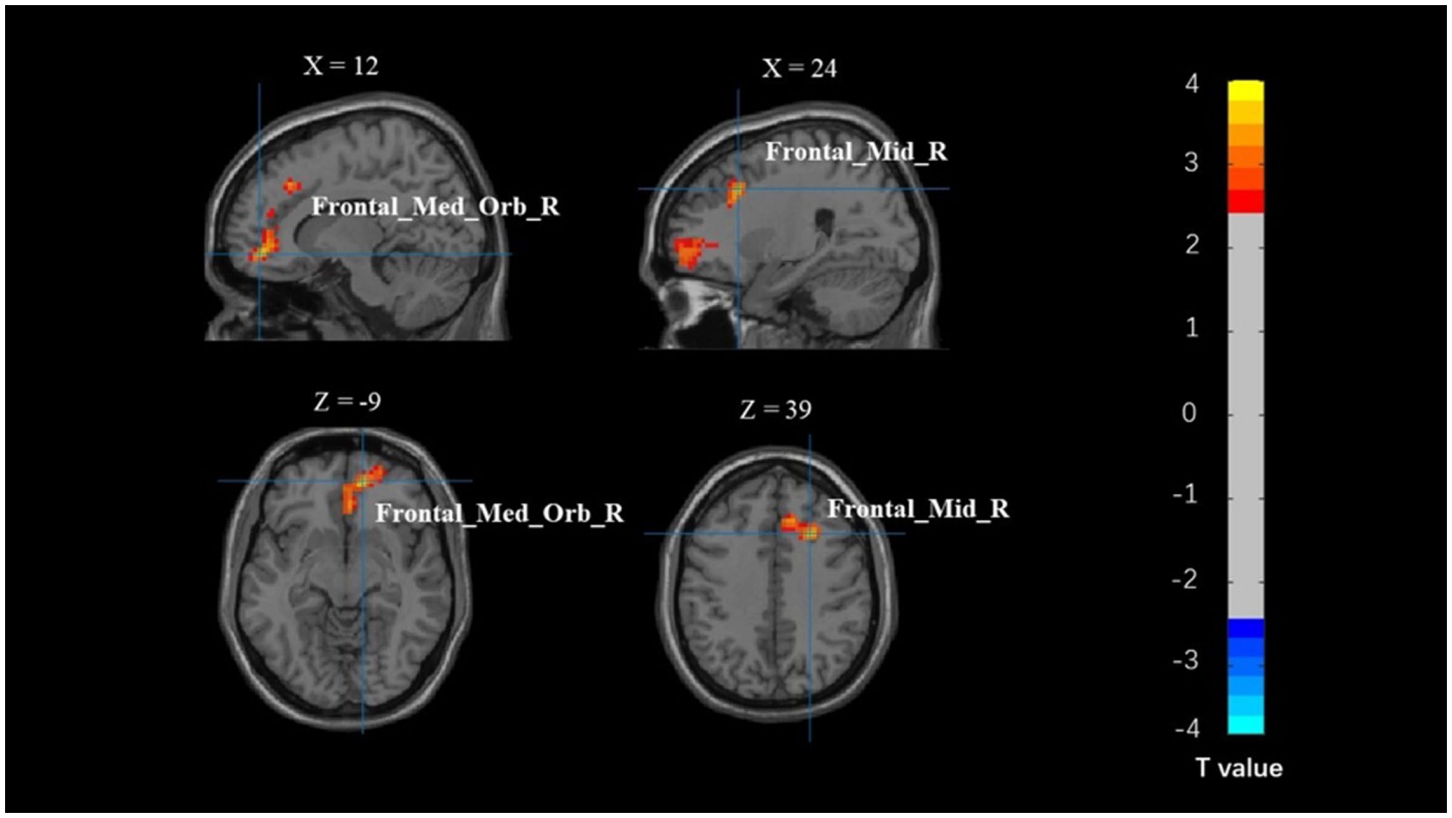
Map of brain activation differences between groups in the incongruent–congruent condition. Frontal_Med_Orb_R, right medial orbitofrontal cortex; Frontal_Mid_R, right middle frontal gyrus.

The whole-brain analysis results from the flanker task for two groups (young adults > 0 and older adults > 0) are presented in Figure 1. In the incongruent vs. congruent condition, compared to the young adults, the older adults showed significantly greater activation in the right medial orbitofrontal cortex (BA 11), right superior orbitofrontal cortex (BA 11), right anterior cingulate cortex (BA 11), right middle frontal gyrus (BA 44), and right superior frontal gyrus (BA 44) (Table 3 and Figure 2).

**Table 3:** Differences in brain activation between age groups (incongruent vs. congruent condition).

Location	Brodmann area	Voxels	Peak *T*	MNI coordinates		
				*X*	*Y*	*Z*
Older adults vs. young adults						
Right medial orbitofrontal cortex	BA11	82	3.9306	12	51	−9
Right superior orbitofrontal cortex	BA11	44	3.9306	12	51	−9
Right anterior cingulate cortex	BA11	36	3.9306	12	51	−9
Right middle frontal gyrus	BA44	91	3.888	24	21	39
Right superior frontal gyrus	BA44	37	3.888	24	21	39

Whole-brain analysis results between two groups (young adults, older adults) with different PA levels are presented in Table 4. Among young adults, the activation in the left inferior frontal gyrus (BA 10), left dorsolateral prefrontal cortex (BA 10), left middle frontal gyrus (BA 10), left middle temporal gyrus (BA 39), and left superior parietal lobule (BA 39) was significantly greater in the physically inactive group compared to the active group. However, there were no statistically significant differences in brain activation between different PA levels among older adults.

**Table 4:** Differences in brain activation across different physical activity levels and age groups.

Location	Brodmann area	Voxels	Peak *T*	MNI coordinates		
				*X*	*Y*	*Z*
Young adults: physically inactive vs. physically active						
Left inferior frontal gyrus	BA10	57	−4.58	−12	54	18
Left dorsolateral prefrontal cortex	BA10	46	−4.58	−12	54	18
Left middle frontal gyrus	BA10	60	−4.58	−12	54	18
Left middle temporal gyrus	BA39	84	−4.32	−42	−54	18
Left superior parietal lobule	BA39	40	−4.32	−42	−54	18
Older adults: physically inactive vs. physically active						
Not significant						

**Figure 3:**
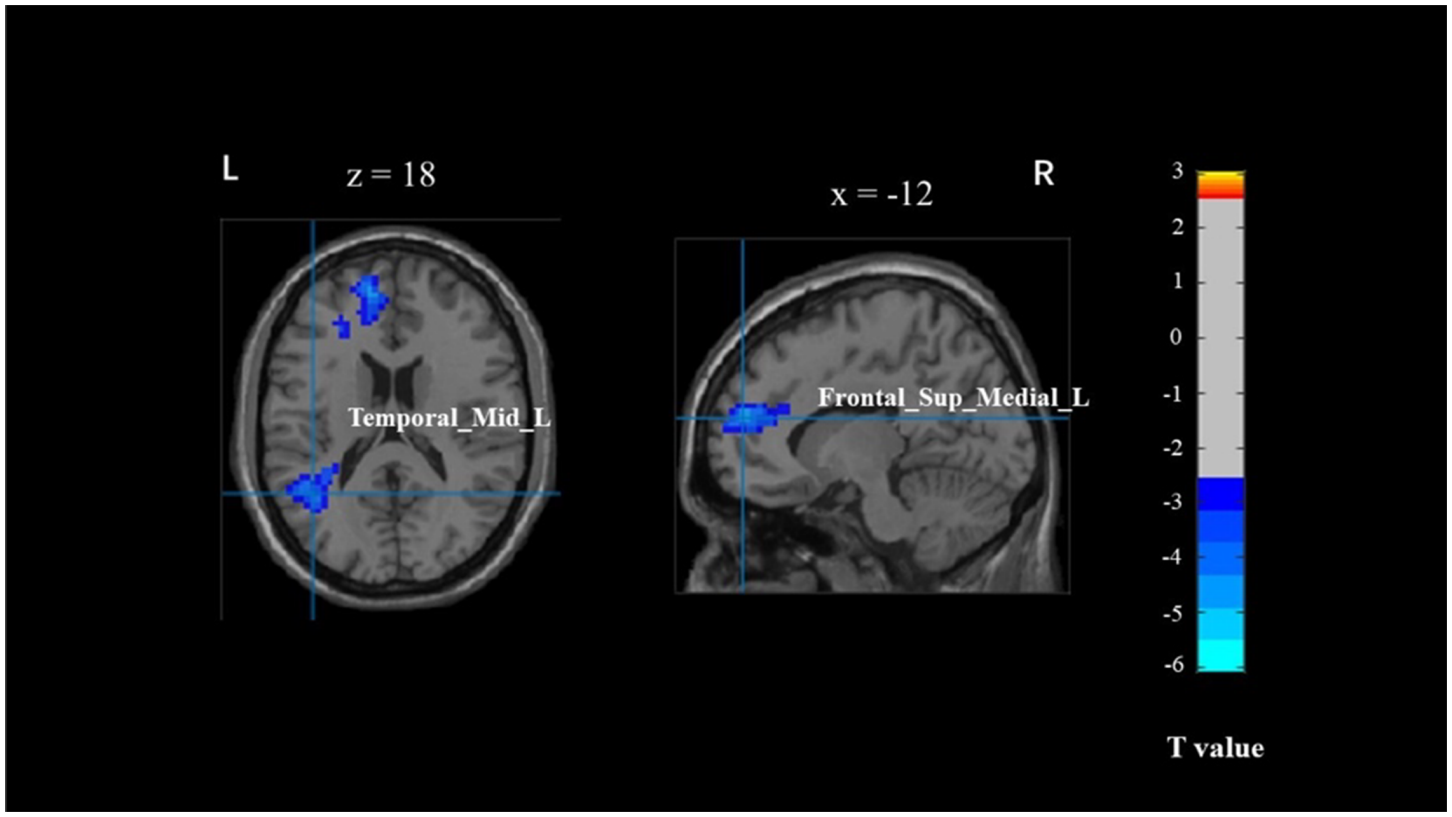
The brain activation associated with behavior across different physical activity levels in young adults. Temporal_Mid_L, left middle temporal gyrus; Frontal_Sup_Medial_L, left superior medial frontal cortex.

The correlation analysis between brain activation and behavioral outcomes across different PA levels (physically inactive, physically active) revealed no significant differences in the brain regions associated with behavior in young adults during the Flanker task, specifically in the left temporal gyrus (BA 39) and left inferior frontal gyrus (BA 10) (Table 5 and Figure 3). There was no correlation observed between brain regions and behavior in older adults.

**Table 5:** The association between brain activation and behavior across different physical activity levels.

	Physically inactive				Physically active			
	Accuracy		Reaction time		Accuracy		Reaction time	
	*r*	*p*	*r*	*p*	*r*	*p*	*r*	*p*
Young adults								
Left temporal gyrus	0.409	0.241	−0.266	0.457	0.354	0.285	−0.160	0.638
Left inferior frontal gyrus	0.041	0.911	−0.043	0.906	−0.163	0.632	0.320	0.338
Older adults								
Nil								

## 4 Discussion

This study examined the relationship of age and PA with cognitive function, as well as its underlying mechanism. The cognitive function of the older adults is significantly worse than that of the young adults. Physically active older adults demonstrate significantly better cognitive function compared to inactive older adults. Compared to active older adults, inactive older adults needs to activate more brain regions to complete the task, specifically in the prefrontal cortex.

The cognitive function of older adults is lower than that of the young adults, indicating an age-related decline. This finding supports the hypothesis of cognitive function decline. Consistent with a study by Hillman et al., young individuals exhibit less reaction times than older adults in the Flanker task (20). Furthermore, higher PA level can be beneficial for the executive function and cognitive processing speed. This study suggests that moderate to high levels of PA may have a protective effect against cognitive decline. Furthermore, this study demonstrates that active older adults exhibits significantly higher inhibitory control abilities compared to the inactive older adults. This is consistent with previous study that suggests a relationship between higher long-term PA and better cognitive inhibition (21). Even during the peak developmental period, regular PA has been shown to have benefits for hemodynamics and cognition in healthy young individuals (21). However, other studies have indicated that MPA, such as dance interventions, are as effective as brisk walking in improving inhibitory control in older adults (22). A study examining the relationship between PA levels (measured using the IPAQ questionnaire and categorized as low, moderate, and high) and individual differences in attention and executive control in the Flanker task among healthy adults (aged 17–45) revealed that the coefficient of reaction time variability was positively correlated with moderate PA levels and negatively correlated with high PA levels (23).

The relationship between PA and inhibitory control was investigated by measuring brain region activation during the Flanker task. Among young adults, compared to the physically active group, the inactive group exhibited higher activation levels in the left medial superior frontal gyrus (BA 10), left superior frontal gyrus (BA 10), left middle frontal gyrus (BA 10), left middle temporal gyrus (BA 39), and left angular gyrus (BA 39), although these differences were not statistically significant. However, there were no significant differences in brain regions between different PA levels in older adults. This suggests that as age increases, the differences in brain activation patterns during inhibitory control tasks diminish between different PA levels. For young adults, the effects of PA are more pronounced, as the inactive group needs to activate more prefrontal brain regions (BA 10, BA 39) to complete the executive control task. This is consistent with the hypothesis of age-related decline in executive functions, where the recruitment of relevant neurons becomes challenging with age (24). Nevertheless, some studies have found that PA can improve the recruitment efficiency of the central nervous system in older adults. For example, a study by Colcombe et al. found that compared to low cardiovascular fitness or control groups, high cardiovascular fitness individuals or aerobic exercise participants exhibited greater activation in the prefrontal and parietal cortices (25). Another study by Yang et al. using functional near-infrared spectroscopy (fNIRS) technology found that 8 weeks of Tai Chi intervention improved performance in the Flanker task and enhanced activation in the left frontal lobe in older adults (26).

In this study, the results of brain region activation related to inhibitory control behavior indicated that different PA levels (physically inactive, physically active) primarily influenced the left middle temporal gyrus (BA 39) and left medial superior frontal gyrus (BA 10) in young adults, but without significant differences in statistical terms. In contrast, there was no statistically significant correlation between brain regions and behavior in older adults. This suggests that as individuals age, the impact of different levels of PA on the brain regions associated with the decline in inhibitory control diminishes and shows no significant effect in older adults. This differs from previous findings. Studies using motion-related fMRI paradigms for executive control functions in children and older adults have found that exercise training interventions primarily target bilateral frontal regions in sedentary middle-aged men during the Flanker task (27). In a comparison of physical fitness levels and executive functions between young and older women using near infrared spectroscopy (NIRS), there is an interaction between physical fitness (high and low) and cognitive abilities (executive conditions, non-executive conditions). Compared to low physical fitness, highly fit women with higher cardiorespiratory fitness exhibit faster reaction times in the Stroop task and higher brain oxygenation in the right inferior frontal gyrus (Right IFG) (28).

This study has several limitations that should be acknowledged. Firstly, due to the nature of the fMRI methodology employed, a considerable number of participants were lost as they were unable to adapt and complete the study. This loss of participants may introduce bias and affect the generalizability of the findings. Secondly, demographic information such as socioeconomic status and retirement status for older participants were not collected in this study. Incorporating these additional participant characteristics could be considered in future research to allow for a more comprehensive understanding of the study population. Thirdly, the cross-sectional study design limits the ability to establish cause-and-effect relationships between PA and inhibitory control. Longitudinal studies and intervention studies would provide a more robust understanding of the relationship, further elucidating the potential causal mechanisms at play.

## 5 Conclusion

The cognitive function of older adults is significantly lower than that of young adults, indicating a decline in the inhibitory control component of executive functions. Physically active older adults demonstrate significantly higher inhibitory control abilities compared to the inactive older adults. Additionally, this study did not find an impact of PA on the activation levels of inhibitory control-related brain regions in older adults but observed benefits of PA on frontal brain regions in young individuals.

## Data availability statement

The raw data supporting the conclusions of this article will be made available by the authors, without undue reservation.

## Ethics statement

The studies involving humans were approved by the Ethical Committee of Shanghai University of Sport. The studies were conducted in accordance with the local legislation and institutional requirements. The participants provided their written informed consent to participate in this study.

## Author contributions

JF: Data curation, Formal analysis, Methodology, Writing – original draft, Writing – review & editing. HS: Data curation, Formal analysis, Methodology, Writing – original draft, Writing – review & editing. YW: Data curation, Writing – original draft, Writing – review & editing. QZ: Formal analysis, Writing – original draft, Writing – review & editing. CZ: Funding acquisition, Writing – original draft, Writing – review & editing. JJ: Conceptualization, Data curation, Formal analysis, Funding acquisition, Investigation, Methodology, Project administration, Resources, Software, Supervision, Validation, Visualization, Writing – original draft, Writing – review & editing.

## Footnotes

^1^http://rfmri.org/DPARSF

^2^http://www.fil.ion.ucl.ac.uk/spm
